# 1-[2-(4-Chloro­benz­yloxy)-2-phenyl­ethyl]-1*H*-benzotriazole

**DOI:** 10.1107/S1600536810015692

**Published:** 2010-05-08

**Authors:** Özden Özel Güven, Meral Bayraktar, Simon J. Coles, Tuncer Hökelek

**Affiliations:** a Department of Chemistry, Zonguldak Karaelmas University, 67100 Zonguldak, Turkey; b Department of Chemistry, Southampton University, Southampton SO17 1BJ, England; c Department of Physics, Hacettepe University, 06800 Beytepe, Ankara, Turkey

## Abstract

The asymmetric unit of the title compound, C_21_H_18_ClN_3_O, contains two crystallographically independent mol­ecules which differ slightly in the orientations of chloro­benz­yloxy units. In one of the mol­ecules, the phenyl and chloro­phenyl rings are oriented at dihedral angles of 38.09 (6) and 42.15 (6)°, respectively, with respect to the benzotriazole ring [43.23 (6) and 29.80 (6)° in the other mol­ecule]. The dihedral angle between the phenyl and chloro­phenyl rings is 77.63 (6)° in one of the mol­ecules and 72.97 (6)° in the other. The crystal structure is stabilized by weak C—H⋯π inter­actions.

## Related literature

For general background to the biological activity of benzotriazole derivatives, see: Hirokawa *et al.* (1998 ▶); Yu *et al.* (2003 ▶); Kopanska *et al.* (2004 ▶). For related structures, see: Caira *et al.* (2004 ▶); Freer *et al.* (1986 ▶); Katritzky *et al.* (2001 ▶); Özel Güven *et al.* (2007*a* ▶,*b* ▶, 2008*a* ▶,*b* ▶,*c* ▶, 2010 ▶); Peeters *et al.* (1979*a* ▶,*b* ▶, 1996 ▶); Swamy *et al.* (2006 ▶).
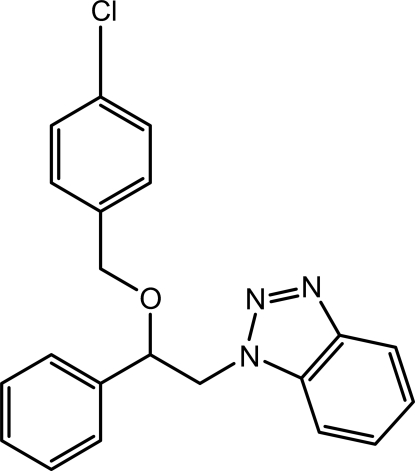


## Experimental

### 

#### Crystal data

C_21_H_18_ClN_3_O
*M* *_r_* = 363.83
Monoclinic, 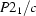

*a* = 7.2163 (2) Å
*b* = 36.8545 (9) Å
*c* = 13.3019 (3) Å
β = 91.529 (1)°
*V* = 3536.42 (15) Å^3^
*Z* = 8
Mo *K*α radiation
μ = 0.23 mm^−1^
*T* = 120 K
0.40 × 0.40 × 0.14 mm

#### Data collection

Nonius Kappa CCD diffractometer
Absorption correction: multi-scan (*SADABS*; Sheldrick, 2007 ▶) *T*_min_ = 0.910, *T*_max_ = 0.966
26731 measured reflections
7993 independent reflections
5918 reflections with *I* > 2σ(*I*)
*R*_int_ = 0.051

#### Refinement

*R*[*F* ^2^ > 2σ(*F* ^2^)] = 0.065
*wR*(*F* ^2^) = 0.189
*S* = 1.07
7993 reflections
470 parameters
H-atom parameters constrained
Δρ_max_ = 0.64 e Å^−3^
Δρ_min_ = −0.69 e Å^−3^

### 

Data collection: *COLLECT* (Nonius, 1998 ▶); cell refinement: *DENZO* (Otwinowski & Minor, 1997 ▶) and *COLLECT*; data reduction: *DENZO* and *COLLECT*; program(s) used to solve structure: *SHELXS97* (Sheldrick, 2008 ▶); program(s) used to refine structure: *SHELXL97* (Sheldrick, 2008 ▶); molecular graphics: *ORTEP-3 for Windows* (Farrugia, 1997 ▶); software used to prepare material for publication: *WinGX* (Farrugia, 1999 ▶) and *PLATON* (Spek, 2009 ▶).

## Supplementary Material

Crystal structure: contains datablocks I, global. DOI: 10.1107/S1600536810015692/ci5085sup1.cif

Structure factors: contains datablocks I. DOI: 10.1107/S1600536810015692/ci5085Isup2.hkl

Additional supplementary materials:  crystallographic information; 3D view; checkCIF report

## Figures and Tables

**Table 1:** Hydrogen-bond geometry (Å, °) *Cg*1, *Cg*2 and *Cg*3 are centroids of the C9–C14, C9′–C14′ and C16–C21 rings, respectively.

*D*—H⋯*A*	*D*—H	H⋯*A*	*D*⋯*A*	*D*—H⋯*A*
C11—H11⋯*Cg*2^i^	0.95	2.62	3.515 (2)	157
C11′—H11′⋯*Cg*1^ii^	0.95	2.79	3.635 (2)	149
C14—H14⋯*Cg*2^iii^	0.95	2.64	3.584 (2)	172
C14′—H14′⋯*Cg*1^iv^	0.95	2.81	3.755 (2)	172
C18′—H18′⋯*Cg*3^v^	0.95	2.83	3.502 (2)	129

Symmetry codes: (i) 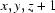; (ii) 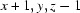; (iii) 
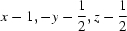
; (iv) 
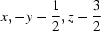
; (v) 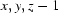.

**Fig. 1.**
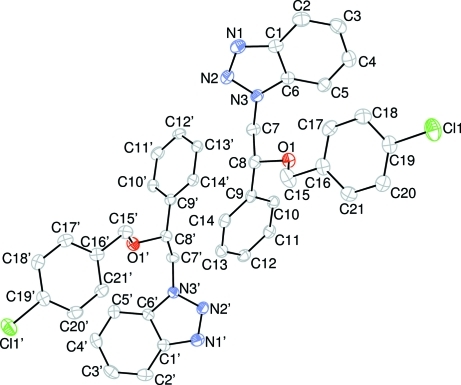
The two independent molecules of the title compound, with the atom-numbering scheme. Displacement ellipsoids are drawn at the 50% probability level. H atoms have been omitted for clarity.

**Fig. 2.**
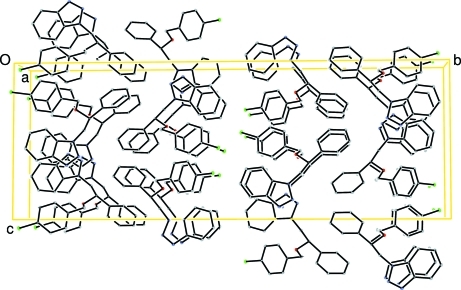
Packing diagram of the title compound, viewed along the a axis. H atoms have been omitted for clarity.

## supplementary crystallographic information

### Comment

In recent years, there has been increasing interest in the syntheses of heterocyclic compounds that have biological and commercial importance. Clotrimazole, miconazole, econazole, ketonazole, itraconazole and fluconazole are well-known imidazoles. 1*H*-1,2,4-Triazole ring containing azole derivatives have been developed for clinical uses as antifungal agents. Recently, structures containing benzimidazole ring in place of miconazole and econazole have been reported, and these molecules have been shown more antibacterial activity than antifungal activity (Özel Güven *et al.*, 2007a,b). The crystal structures of miconazole (Peeters *et al.*, 1979a), ketonazole (Peeters *et al.*, 1979b), econazole (Freer *et al.*, 1986), itraconazole (Peeters *et al.*, 1996) and fluconazole (Caira *et al.*, 2004) have been reported. Recently, we reported crystal structures of related compounds (Özel Güven *et al.*, 2008a,b,c). Benzotriazole derivatives also exhibit a good degree of analgesic, antiinflammatory, diuretic, antiviral and antihypertensive activities (Hirokawa *et al.*, 1998; Yu *et al.*, 2003; Kopanska *et al.*, 2004). Crystal structures of benzotriazole ring possessing compounds have been reported (Katritzky *et al.*, 2001; Swamy *et al.*, 2006; Özel Güven *et al.*, 2010). Now, we report herein the crystal structure of the title benzotriazole derivative.

The asymmetric unit of the title compound (Fig. 1)  contains two crystallographically independent molecules which differ slightly in the orientations of the chlorobenzyloxy units. The bond lengths and angles are generally within normal ranges. In each molecule, the planar benzotriazole rings [A (N1-N3/C1-C6) and A' (N1'-N3'/C1'-C6')] are oriented with respect to the phenyl [B (C9-C14) and B' (C9'-C14')] and benzene [C (C16-C21)] and C' (C16'-C21')] rings at dihedral angles of A/B = 37.98 (9), A/C = 42.27 (9) ° and A'/B' = 43.20 (9), A'/C' = 29.82 (9)°. The dihedral angles between phenyl and benzene rings are B/C = 77.63 (6) and B'/C' = 72.97 (6) °. Atoms C7 and C7' are -0.012 (2) and -0.112 (2) Å away from the planes of the benzotriazole rings, respectively.

In the crystal structure, molecules are stacked along the a-axis (Fig. 2). Weak C—H···π interactions (Table 1) involving the  C9-C14, C9'-C14' and C16-C21 rings stabilize the structure.

### Experimental

The title compound was synthesized by the reaction of 2-(1*H*-benzotriazol-1-yl)-1-phenylethanol (Özel Güven *et al.*, 2010) with aryl halide using NaH. 2-(1*H*-Benzotriazol-1-yl)-1-phenylethanol (200 mg, 0.84 mmol) was dissolved in DMF (4 ml). NaH (33 mg, 0.84 mmol) was added to the solution portionwise. After stirring the mixture a few minutes, 4-chlorobenzyl bromide (171.8 mg, 0.84 mmol) was added dropwise. Then, the reaction mixture was stirred additional 3 h at room temperature. The reaction was stopped by adding methanol (5 ml). After evaporation of the solvent, dichloromethane was added to the reaction mixture and extracted with water. Then, the organic phase was separated, dried, filtered and evaporated. The precipitate formed was purified by column chromatography using chloroform and crystallized from *iso*-propanol to obtain colorless crystals suitable for X-ray analysis (yield; 137 mg, 46%).

### Refinement

H atoms were positioned geometrically with C-H = 0.95, 1.00 and 0.99 Å for aromatic, methine and methylene H, respectively, and constrained to ride on their parent atoms, with U_iso_(H) = 1.2U_eq_(C).

### Crystal data

**Table:**

C_21_H_18_ClN_3_O	*F*(000) = 1520
*M**_r_* = 363.83	*D*_x_ = 1.367 Mg m^−^^3^
Monoclinic, *P*2_1_/*c*	Mo *K*α radiation, λ = 0.71073 Å
Hall symbol: -P 2ybc	Cell parameters from 26277 reflections
*a* = 7.2163 (2) Å	θ = 2.9–27.5°
*b* = 36.8545 (9) Å	µ = 0.23 mm^−^^1^
*c* = 13.3019 (3) Å	*T* = 120 K
β = 91.529 (1)°	Slab, colourless
*V* = 3536.42 (15) Å^3^	0.40 × 0.40 × 0.14 mm
*Z* = 8	

### Data collection

**Table:**

Nonius Kappa CCD diffractometer	7993 independent reflections
Radiation source: fine-focus sealed tube	5918 reflections with *I* > 2σ(*I*)
graphite	*R*_int_ = 0.051
φ and ω scans	θ_max_ = 27.6°, θ_min_ = 3.0°
Absorption correction: multi-scan (*SADABS*; Sheldrick, 2007)	*h* = −9→9
*T*_min_ = 0.910, *T*_max_ = 0.966	*k* = −47→43
26731 measured reflections	*l* = −17→17

### Refinement

**Table:**

Refinement on *F*^2^	Secondary atom site location: difference Fourier map
Least-squares matrix: full	Hydrogen site location: inferred from neighbouring sites
*R*[*F*^2^ > 2σ(*F*^2^)] = 0.065	H-atom parameters constrained
*wR*(*F*^2^) = 0.189	*w* = 1/[σ^2^(*F*_o_^2^) + (0.1171*P*)^2^ + 0.2599*P*] where *P* = (*F*_o_^2^ + 2*F*_c_^2^)/3
*S* = 1.07	(Δ/σ)_max_ = 0.001
7993 reflections	Δρ_max_ = 0.64 e Å^−^^3^
470 parameters	Δρ_min_ = −0.69 e Å^−^^3^
0 restraints	Extinction correction: *SHELXL97* (Sheldrick, 2008), Fc^*^=kFc[1+0.001xFc^2^λ^3^/sin(2θ)]^-1/4^
Primary atom site location: structure-invariant direct methods	Extinction coefficient: 0.038 (2)

### Special details

**Table:**

Geometry. All esds (except the esd in the dihedral angle between two l.s. planes) are estimated using the full covariance matrix. The cell esds are taken into account individually in the estimation of esds in distances, angles and torsion angles; correlations between esds in cell parameters are only used when they are defined by crystal symmetry. An approximate (isotropic) treatment of cell esds is used for estimating esds involving l.s. planes.
Refinement. Refinement of F^2^ against ALL reflections. The weighted R-factor wR and goodness of fit S are based on F^2^, conventional R-factors R are based on F, with F set to zero for negative F^2^. The threshold expression of F^2^ > 2sigma(F^2^) is used only for calculating R-factors(gt) etc. and is not relevant to the choice of reflections for refinement. R-factors based on F^2^ are statistically about twice as large as those based on F, and R- factors based on ALL data will be even larger.

### Fractional atomic coordinates and isotropic or equivalent isotropic displacement parameters (Å^2^)

**Table:**

	*x*	*y*	*z*	*U*_iso_*/*U*_eq_	
Cl1	−0.22475 (9)	0.476481 (18)	0.55416 (6)	0.0416 (2)	
O1	0.3382 (2)	0.34725 (4)	0.40142 (11)	0.0230 (3)	
N1	0.3727 (3)	0.38511 (5)	0.07784 (14)	0.0288 (5)	
N2	0.4375 (3)	0.35556 (5)	0.12052 (14)	0.0269 (4)	
N3	0.4929 (3)	0.36393 (5)	0.21634 (13)	0.0228 (4)	
C1	0.3846 (3)	0.41288 (6)	0.14623 (17)	0.0250 (5)	
C2	0.3361 (3)	0.44961 (7)	0.13830 (19)	0.0327 (6)	
H2	0.2799	0.4591	0.0785	0.039*	
C3	0.3729 (3)	0.47135 (7)	0.2200 (2)	0.0330 (6)	
H3	0.3418	0.4964	0.2166	0.040*	
C4	0.4559 (3)	0.45739 (7)	0.30917 (19)	0.0313 (5)	
H4	0.4821	0.4734	0.3636	0.038*	
C5	0.4999 (3)	0.42131 (6)	0.31946 (17)	0.0264 (5)	
H5	0.5527	0.4118	0.3801	0.032*	
C6	0.4626 (3)	0.39944 (6)	0.23575 (16)	0.0222 (5)	
C7	0.5705 (3)	0.33638 (6)	0.28229 (17)	0.0250 (5)	
H7A	0.6674	0.3474	0.3266	0.030*	
H7B	0.6299	0.3174	0.2414	0.030*	
C8	0.4235 (3)	0.31891 (6)	0.34680 (16)	0.0239 (5)	
H8	0.3278	0.3074	0.3014	0.029*	
C9	0.5062 (3)	0.29013 (6)	0.41518 (15)	0.0209 (5)	
C10	0.6548 (3)	0.29873 (6)	0.48017 (16)	0.0234 (5)	
H10	0.7022	0.3228	0.4821	0.028*	
C11	0.7333 (3)	0.27231 (6)	0.54186 (16)	0.0259 (5)	
H11	0.8358	0.2782	0.5851	0.031*	
C12	0.6629 (3)	0.23736 (6)	0.54066 (16)	0.0254 (5)	
H12	0.7166	0.2193	0.5833	0.030*	
C13	0.5145 (3)	0.22873 (6)	0.47732 (16)	0.0248 (5)	
H13	0.4654	0.2048	0.4769	0.030*	
C14	0.4368 (3)	0.25505 (6)	0.41408 (16)	0.0233 (5)	
H14	0.3359	0.2489	0.3700	0.028*	
C15	0.1497 (3)	0.33957 (7)	0.4250 (2)	0.0360 (6)	
H15A	0.1459	0.3212	0.4791	0.043*	
H15B	0.0831	0.3297	0.3649	0.043*	
C16	0.0583 (3)	0.37382 (6)	0.45829 (18)	0.0265 (5)	
C17	0.0463 (3)	0.40358 (7)	0.39399 (17)	0.0273 (5)	
H17	0.0966	0.4020	0.3288	0.033*	
C18	−0.0374 (3)	0.43532 (6)	0.42360 (18)	0.0291 (5)	
H18	−0.0430	0.4557	0.3799	0.035*	
C19	−0.1137 (3)	0.43699 (6)	0.51846 (18)	0.0278 (5)	
C20	−0.1040 (3)	0.40812 (6)	0.58310 (18)	0.0276 (5)	
H20	−0.1553	0.4097	0.6480	0.033*	
C21	−0.0178 (3)	0.37633 (6)	0.55244 (18)	0.0272 (5)	
H21	−0.0113	0.3561	0.5967	0.033*	
Cl1'	0.19246 (9)	0.026883 (16)	0.22027 (5)	0.0384 (2)	
O1'	0.8366 (2)	0.15017 (4)	0.30657 (11)	0.0248 (4)	
N1'	0.9026 (3)	0.12718 (5)	0.65649 (14)	0.0294 (5)	
N2'	0.9650 (3)	0.15280 (5)	0.59942 (14)	0.0274 (4)	
N3'	0.9881 (3)	0.13952 (5)	0.50553 (13)	0.0225 (4)	
C1'	0.8871 (3)	0.09601 (6)	0.59982 (17)	0.0247 (5)	
C2'	0.8299 (3)	0.06087 (7)	0.62520 (19)	0.0307 (5)	
H2'	0.7928	0.0551	0.6913	0.037*	
C3'	0.8297 (3)	0.03530 (6)	0.5510 (2)	0.0320 (6)	
H3'	0.7897	0.0114	0.5658	0.038*	
C4'	0.8873 (3)	0.04340 (6)	0.45265 (19)	0.0296 (5)	
H4'	0.8868	0.0247	0.4035	0.036*	
C5'	0.9438 (3)	0.07756 (6)	0.42656 (17)	0.0253 (5)	
H5'	0.9818	0.0831	0.3605	0.030*	
C6'	0.9424 (3)	0.10373 (6)	0.50228 (16)	0.0211 (4)	
C7'	1.0704 (3)	0.16200 (6)	0.42962 (16)	0.0235 (5)	
H7C	1.1558	0.1471	0.3898	0.028*	
H7D	1.1442	0.1814	0.4629	0.028*	
C8'	0.9260 (3)	0.17904 (6)	0.35944 (16)	0.0226 (5)	
H8'	0.8322	0.1917	0.4010	0.027*	
C9'	1.0112 (3)	0.20660 (6)	0.29009 (15)	0.0207 (4)	
C10'	1.1563 (3)	0.19730 (6)	0.22767 (17)	0.0241 (5)	
H10'	1.2023	0.1731	0.2278	0.029*	
C11'	1.2337 (3)	0.22296 (7)	0.16565 (17)	0.0290 (5)	
H11'	1.3332	0.2165	0.1239	0.035*	
C12'	1.1663 (3)	0.25810 (6)	0.16425 (16)	0.0262 (5)	
H12'	1.2187	0.2757	0.1212	0.031*	
C13'	1.0226 (3)	0.26762 (6)	0.22557 (16)	0.0243 (5)	
H13'	0.9763	0.2917	0.2244	0.029*	
C14'	0.9455 (3)	0.24203 (6)	0.28896 (16)	0.0228 (5)	
H14'	0.8477	0.2488	0.3316	0.027*	
C15'	0.6640 (3)	0.16060 (6)	0.25888 (18)	0.0287 (5)	
H15C	0.6864	0.1714	0.1922	0.034*	
H15D	0.6006	0.1788	0.3005	0.034*	
C16'	0.5460 (3)	0.12733 (6)	0.24718 (17)	0.0246 (5)	
C17'	0.4741 (3)	0.11645 (7)	0.15453 (17)	0.0265 (5)	
H17'	0.4985	0.1305	0.0965	0.032*	
C18'	0.3671 (3)	0.08540 (6)	0.14502 (18)	0.0267 (5)	
H18'	0.3189	0.0780	0.0811	0.032*	
C19'	0.3319 (3)	0.06545 (6)	0.23027 (18)	0.0272 (5)	
C20'	0.4002 (3)	0.07576 (7)	0.32357 (18)	0.0303 (5)	
H20'	0.3738	0.0617	0.3814	0.036*	
C21'	0.5076 (3)	0.10674 (7)	0.33240 (17)	0.0268 (5)	
H21'	0.5553	0.1140	0.3965	0.032*	

### Atomic displacement parameters (Å^2^)

**Table:**

	*U*^11^	*U*^22^	*U*^33^	*U*^12^	*U*^13^	*U*^23^
Cl1	0.0341 (4)	0.0352 (4)	0.0555 (5)	0.0105 (3)	0.0044 (3)	−0.0115 (3)
O1	0.0185 (8)	0.0235 (8)	0.0271 (8)	−0.0002 (6)	0.0016 (6)	−0.0028 (6)
N1	0.0275 (11)	0.0344 (11)	0.0244 (10)	0.0009 (9)	−0.0033 (8)	0.0026 (9)
N2	0.0290 (11)	0.0310 (11)	0.0205 (10)	−0.0007 (8)	−0.0004 (8)	−0.0020 (8)
N3	0.0245 (10)	0.0243 (9)	0.0197 (9)	0.0029 (7)	0.0006 (7)	0.0003 (7)
C1	0.0201 (11)	0.0300 (12)	0.0249 (11)	−0.0002 (9)	0.0007 (9)	0.0042 (9)
C2	0.0273 (13)	0.0369 (14)	0.0339 (13)	0.0034 (10)	−0.0004 (10)	0.0076 (11)
C3	0.0301 (13)	0.0263 (12)	0.0428 (15)	0.0051 (10)	0.0040 (11)	0.0040 (11)
C4	0.0260 (12)	0.0310 (13)	0.0371 (14)	−0.0006 (10)	0.0045 (10)	−0.0057 (11)
C5	0.0234 (12)	0.0318 (12)	0.0241 (11)	−0.0007 (9)	0.0035 (9)	−0.0004 (9)
C6	0.0182 (10)	0.0247 (11)	0.0239 (11)	0.0010 (9)	0.0018 (9)	0.0026 (9)
C7	0.0236 (11)	0.0264 (11)	0.0252 (11)	0.0053 (9)	0.0034 (9)	0.0025 (9)
C8	0.0219 (11)	0.0268 (11)	0.0229 (11)	0.0025 (9)	0.0012 (9)	−0.0021 (9)
C9	0.0185 (11)	0.0250 (11)	0.0193 (10)	0.0029 (8)	0.0031 (8)	−0.0047 (9)
C10	0.0246 (11)	0.0241 (11)	0.0215 (11)	0.0014 (9)	0.0025 (9)	−0.0010 (9)
C11	0.0256 (12)	0.0306 (12)	0.0215 (11)	0.0004 (9)	−0.0023 (9)	−0.0023 (9)
C12	0.0280 (12)	0.0278 (12)	0.0205 (11)	0.0059 (9)	0.0038 (9)	0.0013 (9)
C13	0.0251 (12)	0.0247 (11)	0.0248 (11)	−0.0002 (9)	0.0058 (9)	−0.0023 (9)
C14	0.0198 (11)	0.0282 (11)	0.0219 (11)	0.0007 (9)	0.0012 (8)	−0.0048 (9)
C15	0.0226 (12)	0.0290 (13)	0.0569 (17)	−0.0013 (10)	0.0100 (11)	−0.0076 (12)
C16	0.0157 (11)	0.0286 (12)	0.0350 (13)	−0.0019 (9)	−0.0002 (9)	−0.0056 (10)
C17	0.0203 (11)	0.0381 (13)	0.0237 (11)	0.0015 (10)	0.0027 (9)	−0.0016 (10)
C18	0.0254 (12)	0.0308 (12)	0.0310 (13)	0.0051 (10)	−0.0034 (10)	0.0038 (10)
C19	0.0186 (11)	0.0300 (12)	0.0348 (13)	0.0012 (9)	−0.0003 (9)	−0.0056 (10)
C20	0.0201 (11)	0.0342 (13)	0.0285 (12)	−0.0028 (9)	0.0032 (9)	−0.0037 (10)
C21	0.0193 (11)	0.0288 (12)	0.0336 (13)	−0.0038 (9)	0.0023 (9)	0.0026 (10)
Cl1'	0.0379 (4)	0.0285 (3)	0.0483 (4)	−0.0063 (3)	−0.0066 (3)	0.0003 (3)
O1'	0.0202 (8)	0.0246 (8)	0.0295 (9)	−0.0014 (6)	−0.0015 (6)	−0.0010 (7)
N1'	0.0327 (11)	0.0292 (10)	0.0267 (10)	0.0066 (9)	0.0044 (8)	0.0024 (8)
N2'	0.0342 (11)	0.0265 (10)	0.0216 (10)	0.0032 (8)	0.0005 (8)	−0.0014 (8)
N3'	0.0248 (10)	0.0205 (9)	0.0220 (9)	−0.0007 (7)	−0.0009 (7)	0.0005 (7)
C1'	0.0231 (11)	0.0235 (11)	0.0275 (12)	0.0040 (9)	0.0028 (9)	0.0016 (9)
C2'	0.0249 (12)	0.0323 (13)	0.0351 (13)	0.0054 (10)	0.0068 (10)	0.0099 (11)
C3'	0.0243 (12)	0.0230 (11)	0.0489 (15)	0.0012 (9)	0.0029 (11)	0.0054 (11)
C4'	0.0264 (12)	0.0226 (11)	0.0398 (14)	0.0019 (9)	−0.0020 (10)	−0.0054 (10)
C5'	0.0222 (11)	0.0261 (12)	0.0276 (12)	0.0023 (9)	−0.0015 (9)	−0.0019 (9)
C6'	0.0180 (10)	0.0200 (10)	0.0253 (11)	0.0013 (8)	−0.0003 (8)	0.0018 (9)
C7'	0.0221 (11)	0.0245 (11)	0.0240 (11)	−0.0042 (9)	−0.0012 (9)	0.0044 (9)
C8'	0.0201 (11)	0.0236 (11)	0.0239 (11)	−0.0009 (9)	−0.0014 (9)	0.0005 (9)
C9'	0.0188 (10)	0.0241 (11)	0.0191 (10)	−0.0010 (8)	−0.0031 (8)	0.0007 (9)
C10'	0.0225 (11)	0.0244 (11)	0.0254 (11)	0.0015 (9)	−0.0003 (9)	−0.0029 (9)
C11'	0.0234 (12)	0.0401 (14)	0.0238 (12)	−0.0014 (10)	0.0050 (9)	−0.0011 (10)
C12'	0.0270 (12)	0.0324 (12)	0.0191 (11)	−0.0086 (10)	−0.0023 (9)	0.0053 (9)
C13'	0.0274 (12)	0.0237 (11)	0.0217 (11)	−0.0026 (9)	−0.0049 (9)	0.0002 (9)
C14'	0.0227 (11)	0.0255 (11)	0.0202 (11)	−0.0002 (9)	−0.0014 (8)	−0.0017 (9)
C15'	0.0226 (12)	0.0334 (13)	0.0298 (12)	−0.0030 (10)	−0.0054 (9)	0.0052 (10)
C16'	0.0166 (10)	0.0295 (12)	0.0277 (12)	0.0022 (9)	0.0008 (9)	0.0035 (10)
C17'	0.0186 (11)	0.0360 (13)	0.0250 (12)	0.0022 (9)	−0.0001 (9)	0.0057 (10)
C18'	0.0211 (11)	0.0325 (12)	0.0264 (11)	0.0035 (9)	−0.0022 (9)	−0.0038 (10)
C19'	0.0210 (11)	0.0260 (11)	0.0346 (13)	0.0010 (9)	−0.0015 (10)	−0.0023 (10)
C20'	0.0265 (12)	0.0382 (14)	0.0264 (12)	−0.0035 (10)	0.0015 (9)	0.0063 (10)
C21'	0.0214 (11)	0.0355 (13)	0.0233 (11)	−0.0013 (9)	−0.0012 (9)	0.0010 (10)

### Geometric parameters (Å, °)

**Table:**

Cl1—C19	1.734 (2)	Cl1'—C19'	1.745 (2)
O1—C8	1.422 (3)	O1'—C8'	1.421 (3)
O1—C15	1.432 (3)	O1'—C15'	1.435 (3)
N1—N2	1.309 (3)	N1'—N2'	1.299 (3)
N1—C1	1.371 (3)	N1'—C1'	1.377 (3)
N2—N3	1.361 (2)	N2'—N3'	1.356 (3)
N3—C6	1.353 (3)	N3'—C6'	1.360 (3)
N3—C7	1.445 (3)	N3'—C7'	1.446 (3)
C1—C6	1.394 (3)	C1'—C6'	1.397 (3)
C1—C2	1.401 (3)	C1'—C2'	1.403 (3)
C2—C3	1.371 (4)	C2'—C3'	1.365 (4)
C2—H2	0.95	C2'—H2'	0.95
C3—C4	1.411 (3)	C3'—C4'	1.415 (3)
C3—H3	0.95	C3'—H3'	0.95
C4—C5	1.373 (3)	C4'—C5'	1.371 (3)
C4—H4	0.95	C4'—H4'	0.95
C5—C6	1.395 (3)	C5'—C6'	1.395 (3)
C5—H5	0.95	C5'—H5'	0.9500
C7—C8	1.525 (3)	C7'—C8'	1.516 (3)
C7—H7A	0.99	C7'—H7C	0.99
C7—H7B	0.99	C7'—H7D	0.99
C8—C9	1.510 (3)	C8'—C9'	1.514 (3)
C8—H8	1.00	C8'—H8'	1.00
C9—C14	1.387 (3)	C9'—C14'	1.389 (3)
C9—C10	1.396 (3)	C9'—C10'	1.397 (3)
C10—C11	1.385 (3)	C10'—C11'	1.382 (3)
C10—H10	0.95	C10'—H10'	0.95
C11—C12	1.385 (3)	C11'—C12'	1.383 (3)
C11—H11	0.95	C11'—H11'	0.95
C12—C13	1.382 (3)	C12'—C13'	1.381 (3)
C12—H12	0.95	C12'—H12'	0.95
C13—C14	1.392 (3)	C13'—C14'	1.391 (3)
C13—H13	0.95	C13'—H13'	0.95
C14—H14	0.95	C14'—H14'	0.95
C15—C16	1.497 (3)	C15'—C16'	1.498 (3)
C15—H15A	0.99	C15'—H15C	0.99
C15—H15B	0.99	C15'—H15D	0.99
C16—C21	1.384 (3)	C16'—C17'	1.384 (3)
C16—C17	1.392 (3)	C16'—C21'	1.398 (3)
C17—C18	1.379 (3)	C17'—C18'	1.384 (3)
C17—H17	0.95	C17'—H17'	0.95
C18—C19	1.391 (3)	C18'—C19'	1.380 (3)
C18—H18	0.95	C18'—H18'	0.95
C19—C20	1.369 (3)	C19'—C20'	1.377 (3)
C20—C21	1.393 (3)	C20'—C21'	1.383 (3)
C20—H20	0.95	C20'—H20'	0.95
C21—H21	0.95	C21'—H21'	0.95
			
C8—O1—C15	113.13 (17)	C8'—O1'—C15'	113.31 (17)
N2—N1—C1	108.55 (18)	N2'—N1'—C1'	108.11 (18)
N1—N2—N3	108.07 (18)	N1'—N2'—N3'	109.20 (18)
C6—N3—N2	110.69 (17)	N2'—N3'—C6'	110.10 (18)
C6—N3—C7	128.75 (18)	N2'—N3'—C7'	119.96 (18)
N2—N3—C7	120.56 (18)	C6'—N3'—C7'	129.59 (19)
N1—C1—C6	108.6 (2)	N1'—C1'—C6'	108.51 (19)
N1—C1—C2	131.3 (2)	N1'—C1'—C2'	131.2 (2)
C6—C1—C2	120.1 (2)	C6'—C1'—C2'	120.3 (2)
C3—C2—C1	117.5 (2)	C3'—C2'—C1'	117.2 (2)
C3—C2—H2	121.2	C3'—C2'—H2'	121.4
C1—C2—H2	121.2	C1'—C2'—H2'	121.4
C2—C3—C4	121.5 (2)	C2'—C3'—C4'	121.9 (2)
C2—C3—H3	119.3	C2'—C3'—H3'	119.0
C4—C3—H3	119.3	C4'—C3'—H3'	119.0
C5—C4—C3	121.9 (2)	C5'—C4'—C3'	121.6 (2)
C5—C4—H4	119.0	C5'—C4'—H4'	119.2
C3—C4—H4	119.0	C3'—C4'—H4'	119.2
C4—C5—C6	116.2 (2)	C4'—C5'—C6'	116.4 (2)
C4—C5—H5	121.9	C4'—C5'—H5'	121.8
C6—C5—H5	121.9	C6'—C5'—H5'	121.8
N3—C6—C1	104.13 (19)	N3'—C6'—C5'	133.4 (2)
N3—C6—C5	133.0 (2)	N3'—C6'—C1'	104.07 (19)
C1—C6—C5	122.8 (2)	C5'—C6'—C1'	122.5 (2)
N3—C7—C8	111.97 (18)	N3'—C7'—C8'	112.26 (18)
N3—C7—H7A	109.2	N3'—C7'—H7C	109.2
C8—C7—H7A	109.2	C8'—C7'—H7C	109.2
N3—C7—H7B	109.2	N3'—C7'—H7D	109.2
C8—C7—H7B	109.2	C8'—C7'—H7D	109.2
H7A—C7—H7B	107.9	H7C—C7'—H7D	107.9
O1—C8—C9	112.19 (17)	O1'—C8'—C9'	112.79 (17)
O1—C8—C7	107.12 (17)	O1'—C8'—C7'	106.87 (17)
C9—C8—C7	111.46 (18)	C9'—C8'—C7'	111.70 (18)
O1—C8—H8	108.7	O1'—C8'—H8'	108.4
C9—C8—H8	108.7	C9'—C8'—H8'	108.4
C7—C8—H8	108.7	C7'—C8'—H8'	108.4
C14—C9—C10	119.3 (2)	C14'—C9'—C10'	119.0 (2)
C14—C9—C8	120.78 (19)	C14'—C9'—C8'	119.57 (19)
C10—C9—C8	119.91 (19)	C10'—C9'—C8'	121.38 (19)
C11—C10—C9	120.2 (2)	C11'—C10'—C9'	120.5 (2)
C11—C10—H10	119.9	C11'—C10'—H10'	119.7
C9—C10—H10	119.9	C9'—C10'—H10'	119.7
C12—C11—C10	120.2 (2)	C10'—C11'—C12'	120.1 (2)
C12—C11—H11	119.9	C10'—C11'—H11'	120.0
C10—C11—H11	119.9	C12'—C11'—H11'	120.0
C13—C12—C11	120.0 (2)	C13'—C12'—C11'	119.9 (2)
C13—C12—H12	120.0	C13'—C12'—H12'	120.0
C11—C12—H12	120.0	C11'—C12'—H12'	120.0
C12—C13—C14	120.1 (2)	C12'—C13'—C14'	120.3 (2)
C12—C13—H13	120.0	C12'—C13'—H13'	119.9
C14—C13—H13	120.0	C14'—C13'—H13'	119.9
C9—C14—C13	120.3 (2)	C9'—C14'—C13'	120.1 (2)
C9—C14—H14	119.9	C9'—C14'—H14'	119.9
C13—C14—H14	119.9	C13'—C14'—H14'	119.9
O1—C15—C16	109.06 (18)	O1'—C15'—C16'	108.11 (18)
O1—C15—H15A	109.9	O1'—C15'—H15C	110.1
C16—C15—H15A	109.9	C16'—C15'—H15C	110.1
O1—C15—H15B	109.9	O1'—C15'—H15D	110.1
C16—C15—H15B	109.9	C16'—C15'—H15D	110.1
H15A—C15—H15B	108.3	H15C—C15'—H15D	108.4
C21—C16—C17	118.9 (2)	C17'—C16'—C21'	119.1 (2)
C21—C16—C15	120.9 (2)	C17'—C16'—C15'	121.9 (2)
C17—C16—C15	120.2 (2)	C21'—C16'—C15'	119.0 (2)
C18—C17—C16	120.9 (2)	C16'—C17'—C18'	121.1 (2)
C18—C17—H17	119.6	C16'—C17'—H17'	119.5
C16—C17—H17	119.6	C18'—C17'—H17'	119.5
C17—C18—C19	118.9 (2)	C19'—C18'—C17'	118.6 (2)
C17—C18—H18	120.5	C19'—C18'—H18'	120.7
C19—C18—H18	120.5	C17'—C18'—H18'	120.7
C20—C19—C18	121.4 (2)	C20'—C19'—C18'	121.6 (2)
C20—C19—Cl1	119.79 (18)	C20'—C19'—Cl1'	119.05 (18)
C18—C19—Cl1	118.85 (19)	C18'—C19'—Cl1'	119.30 (18)
C19—C20—C21	119.1 (2)	C19'—C20'—C21'	119.4 (2)
C19—C20—H20	120.5	C19'—C20'—H20'	120.3
C21—C20—H20	120.5	C21'—C20'—H20'	120.3
C16—C21—C20	120.8 (2)	C20'—C21'—C16'	120.1 (2)
C16—C21—H21	119.6	C20'—C21'—H21'	119.9
C20—C21—H21	119.6	C16'—C21'—H21'	119.9
			
C1—N1—N2—N3	−0.4 (2)	C1'—N1'—N2'—N3'	1.1 (2)
N1—N2—N3—C6	0.2 (2)	N1'—N2'—N3'—C6'	−1.2 (2)
N1—N2—N3—C7	179.79 (19)	N1'—N2'—N3'—C7'	−175.07 (19)
N2—N1—C1—C6	0.4 (3)	N2'—N1'—C1'—C6'	−0.6 (3)
N2—N1—C1—C2	179.3 (2)	N2'—N1'—C1'—C2'	178.9 (2)
N1—C1—C2—C3	−177.2 (2)	N1'—C1'—C2'—C3'	−179.9 (2)
C6—C1—C2—C3	1.7 (3)	C6'—C1'—C2'—C3'	−0.5 (3)
C1—C2—C3—C4	−0.2 (4)	C1'—C2'—C3'—C4'	1.0 (4)
C2—C3—C4—C5	−1.6 (4)	C2'—C3'—C4'—C5'	−1.0 (4)
C3—C4—C5—C6	1.7 (3)	C3'—C4'—C5'—C6'	0.4 (3)
N2—N3—C6—C1	0.1 (2)	N2'—N3'—C6'—C5'	−178.8 (2)
C7—N3—C6—C1	−179.5 (2)	C7'—N3'—C6'—C5'	−5.7 (4)
N2—N3—C6—C5	−177.4 (2)	N2'—N3'—C6'—C1'	0.8 (2)
C7—N3—C6—C5	3.0 (4)	C7'—N3'—C6'—C1'	173.9 (2)
N1—C1—C6—N3	−0.3 (2)	C4'—C5'—C6'—N3'	179.7 (2)
C2—C1—C6—N3	−179.4 (2)	C4'—C5'—C6'—C1'	0.1 (3)
N1—C1—C6—C5	177.6 (2)	N1'—C1'—C6'—N3'	−0.2 (2)
C2—C1—C6—C5	−1.5 (3)	C2'—C1'—C6'—N3'	−179.7 (2)
C4—C5—C6—N3	176.9 (2)	N1'—C1'—C6'—C5'	179.5 (2)
C4—C5—C6—C1	−0.2 (3)	C2'—C1'—C6'—C5'	0.0 (3)
C6—N3—C7—C8	85.9 (3)	N2'—N3'—C7'—C8'	−99.2 (2)
N2—N3—C7—C8	−93.6 (2)	C6'—N3'—C7'—C8'	88.3 (3)
C15—O1—C8—C9	−86.2 (2)	C15'—O1'—C8'—C9'	−73.5 (2)
C15—O1—C8—C7	151.18 (19)	C15'—O1'—C8'—C7'	163.40 (18)
N3—C7—C8—O1	−57.0 (2)	N3'—C7'—C8'—O1'	−64.4 (2)
N3—C7—C8—C9	179.89 (17)	N3'—C7'—C8'—C9'	171.82 (18)
O1—C8—C9—C14	115.0 (2)	O1'—C8'—C9'—C14'	116.4 (2)
C7—C8—C9—C14	−124.9 (2)	C7'—C8'—C9'—C14'	−123.2 (2)
O1—C8—C9—C10	−65.3 (2)	O1'—C8'—C9'—C10'	−64.3 (3)
C7—C8—C9—C10	54.9 (3)	C7'—C8'—C9'—C10'	56.1 (3)
C14—C9—C10—C11	0.8 (3)	C14'—C9'—C10'—C11'	0.0 (3)
C8—C9—C10—C11	−178.91 (19)	C8'—C9'—C10'—C11'	−179.3 (2)
C9—C10—C11—C12	−1.1 (3)	C9'—C10'—C11'—C12'	−0.6 (3)
C10—C11—C12—C13	0.4 (3)	C10'—C11'—C12'—C13'	0.6 (3)
C11—C12—C13—C14	0.6 (3)	C11'—C12'—C13'—C14'	0.1 (3)
C10—C9—C14—C13	0.1 (3)	C10'—C9'—C14'—C13'	0.7 (3)
C8—C9—C14—C13	179.87 (19)	C8'—C9'—C14'—C13'	179.99 (19)
C12—C13—C14—C9	−0.8 (3)	C12'—C13'—C14'—C9'	−0.8 (3)
C8—O1—C15—C16	−165.84 (19)	C8'—O1'—C15'—C16'	−154.90 (18)
O1—C15—C16—C21	−121.1 (2)	O1'—C15'—C16'—C17'	−124.1 (2)
O1—C15—C16—C17	59.8 (3)	O1'—C15'—C16'—C21'	55.9 (3)
C21—C16—C17—C18	0.9 (3)	C21'—C16'—C17'—C18'	−0.8 (3)
C15—C16—C17—C18	180.0 (2)	C15'—C16'—C17'—C18'	179.2 (2)
C16—C17—C18—C19	−1.3 (4)	C16'—C17'—C18'—C19'	0.5 (3)
C17—C18—C19—C20	1.2 (4)	C17'—C18'—C19'—C20'	0.1 (3)
C17—C18—C19—Cl1	−178.19 (18)	C17'—C18'—C19'—Cl1'	178.37 (17)
C18—C19—C20—C21	−0.8 (4)	C18'—C19'—C20'—C21'	−0.3 (4)
Cl1—C19—C20—C21	178.61 (17)	Cl1'—C19'—C20'—C21'	−178.62 (18)
C17—C16—C21—C20	−0.5 (3)	C19'—C20'—C21'—C16'	0.0 (4)
C15—C16—C21—C20	−179.5 (2)	C17'—C16'—C21'—C20'	0.5 (3)
C19—C20—C21—C16	0.4 (3)	C15'—C16'—C21'—C20'	−179.5 (2)

### Hydrogen-bond geometry (Å, °)

**Table:**

Cg1, Cg2 and Cg3 are centroids of the C9–C14, C9'–C14' and C16–C21 rings, respectively.

**Table:**

*D*—H···*A*	*D*—H	H···*A*	*D*···*A*	*D*—H···*A*
C11—H11···Cg2^i^	0.95	2.62	3.515 (2)	157
C11'—H11'···Cg1^ii^	0.95	2.79	3.635 (2)	149
C14—H14···Cg2^iii^	0.95	2.64	3.584 (2)	172
C14'—H14'···Cg1^iv^	0.95	2.81	3.755 (2)	172
C18'—H18'···Cg3^v^	0.95	2.83	3.502 (2)	129

Symmetry codes: (i) *x*, *y*, *z*+1; (ii) *x*+1, *y*, *z*−1; (iii) *x*−1, −*y*−1/2, *z*−1/2; (iv) *x*, −*y*−1/2, *z*−3/2; (v) *x*, *y*, *z*−1.
